# Surgical Management of Myringosclerosis over an Entire Perforated Tympanic Membrane by Simple Underlay Myringoplasty

**DOI:** 10.1155/2016/2894932

**Published:** 2016-06-30

**Authors:** Masayuki Furukawa, Chieri Hayashi, Osamu Narabayashi, Misato Kasai, Hiroko Okada, Takuo Haruyama, Akira Minekawa, Takashi Iizuka, Katsuhisa Ikeda

**Affiliations:** Department of Otorhinolaryngology, Juntendo University School of Medicine, 2-1-1 Hongo, Bunkyo-ku, Tokyo 113-8421, Japan

## Abstract

The aim of our study is to demonstrate the surgical management of myringosclerosis over a perforated whole tympanic membrane using simple underlay myringoplasty. Simple underlay myringoplasty with fibrin glue was performed in 11 ears with myringosclerosis over the entire tympanic membrane. The patients were one male and ten females and their mean age was 61.8 years (range, 40–73 yr). Surgical success was defined as an intact tympanic membrane 12 months after surgery. Closure of the perforation was successful in 10 (91%) of the 11 patients. Failure of the graft occurred in one patient who then underwent a revision procedure using her stored fascia in the outpatient clinic with a successful outcome. The overall success rate was 100%. Although this study included a small number of cases, removal of myringosclerosis at the edge of a perforation is a beneficial technique for simple underlay myringoplasty in terms of the success rate and postoperative hearing threshold, especially when myringosclerosis extends over the entire tympanic membrane.

## 1. Introduction

Myringosclerosis is a pathologic condition affecting the tympanic membrane. It appears as whitish, sclerotic plaques in certain areas of the tympanic membrane. Histologically, there is an increase in collagen fibers as well as hyaline degeneration within the lamina propria [1–3]. The pathogenesis of myringosclerosis is complex and cannot be explained by any single factor, but associated factors include otitis media with effusion treated with myringotomy [4] and tympanostomy tubes [5, 6]. Myringosclerosis caused by insertion of the tympanostomy tubes is not known to affect the hearing threshold [7]. Myringosclerosis is considered a healed inflammation or a particular form of scar tissue following recurrent otitis media. Myringosclerosis is also seen in association with perforated tympanic membrane. Both myringosclerosis and blood vessels exist in the lamina propria of the tympanic membrane [8]. Thus, removing the myringosclerosis over a perforated whole tympanic membrane might influence the grafting success.

In Japan, simple underlay myringoplasty with fibrin glue is a well-established procedure because of its high success rate and low risk of sensorineural hearing loss [9–11]. The aim of our study is to demonstrate the surgical management of myringosclerosis over a perforated whole tympanic membrane using simple underlay myringoplasty.

## 2. Case Presentation

**Figure 1:**
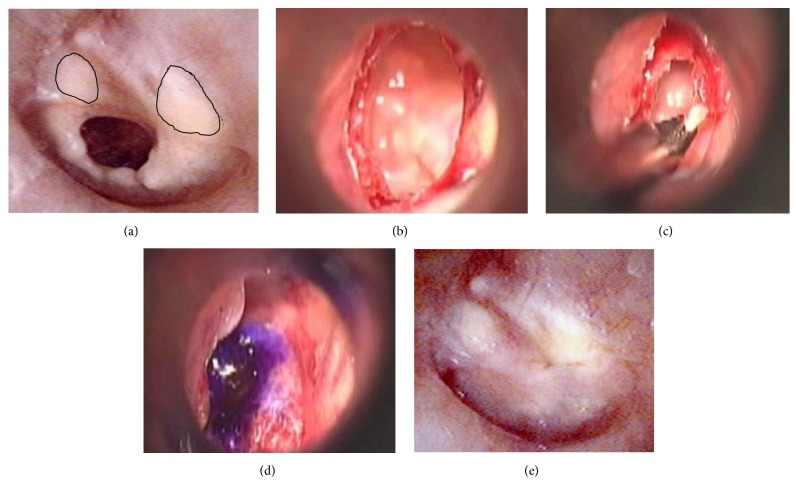
Operative procedures of simple underlay myringoplasty in a representative case with myringosclerosis. (a) The preoperative perforated tympanic membrane with myringosclerosis (the area surrounded by solid lines). (b) The refreshened edge of the perforation. (c) Removal of myringosclerotic deposits. (d) The underlay graft with marked purple. (e) The postoperative finding of the tympanic membrane.

We present our operative procedure of simple underlay myringoplasty with myringosclerosis (Figure 1(a)) over the whole tympanic membrane under general anesthesia. The indications for surgery include hearing improvement after closure of the perforation by paper patch, dry ear at operation, and wide/straight external ear canal. First, the edge of the perforation is removed with a fine pick through an ear speculum to create a vascular bed (Figure 1(b)). Secondly, myringosclerotic deposits at the edge of the perforation are removed from the lamina propria, while preserving the epidermis and the mucosal layer as much as possible (Figure 1(c)). It is impossible to remove the myringosclerotic deposits all at once from the lamina propria at the edge of the perforation. The myringosclerotic deposits are removed from the lamina propria on all sides of the perforation stepwise with a fine pick, straight microforceps, curved right microforceps, curved left microforceps, and microscissors. Although myringosclerosis still remains in the anterior superior and posterior superior quadrants, no myringosclerosis is observed at the edge of the perforation. Finally, fascia obtained from the retroauricular region is fixed as an underlay with a few drops of fibrin glue (Figure 1(d)). An endoscope is employed for patients in whom the margin of the perforation of the tympanic membrane is invisible with the operating microscope due to a curved external auditory canal. A portion of the remaining fascia is kept in a deep freezer to be used for repairing any perforation in the postoperative course. The repair procedure is performed in the same way as the simple underlay myringoplasty. The main points of the surgical technique are presented again with schematic drawings. At the edge of the perforation, the epidermis not only covers the edge, but also extends beneath the tympanic membrane. The edge of the perforation is cut with a fine pick. After removal of the epidermis which extends to the mucous layer, the myringosclerotic deposits are removed from the lamina propria, while preserving the epidermis and the mucous layer as much as possible. Myringosclerosis still remains beyond the edge of the perforation. The fascia is fixed as an underlay graft with fibrin glue. After several months, the tympanic membrane is intact despite the remaining myringosclerosis in the anterior superior and posterior quadrants (Figure 1(e)).

**Figure 2:**
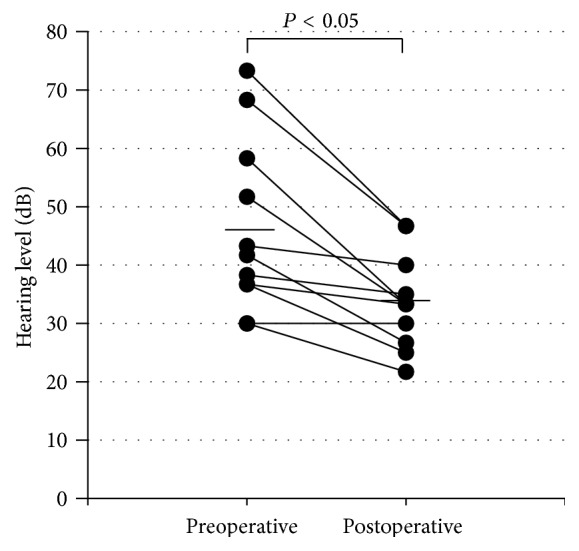
Scatterplots of hearing levels at preoperative and postoperative stages. Significant improvement of hearing level is recognized after surgery. Horizontal bars denote averages for each stage.

**Figure 3:**
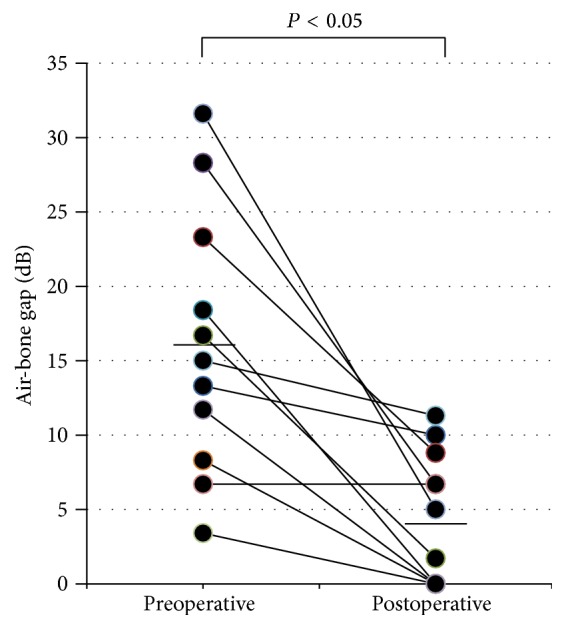
Scatterplots of air-bone gaps at preoperative and postoperative stages. Significant reduction is observed after surgery. Horizontal bars denote averages for each stage.

Closure of the perforation was successful in 10 (91%) of the 11 patients. Failure of the graft occurred in one patient who underwent a revision procedure using her stored fascia in the outpatient clinic with a successful outcome. The overall success rate was 100%. The air conduction hearing level postoperatively was significantly improved as compared with that of preoperative level (Figure 2). The air-bone gap was also significantly (one-way ANOVA) reduced after surgery (Figure 3). Hearing gain after operation was 12.4 ± 8.7 dB (mean ± standard deviation).

All patients gave their written informed consent, and the study was approved by the ethics committee of Juntendo University Faculty of Medicine.

## 3. Discussion

Myringosclerosis is the term used to describe hyalinization and calcification of the connective tissue layer in the lamina propria of the tympanic membrane. The exact etiology and pathogenesis of myringosclerosis are not known. Myringosclerosis often occurs in patients who had undergone the insertion of tympanostomy tubes. An increased oxygen concentration in the atmosphere of the ear is associated with the increased development of myringosclerosis in a traumatized tympanic membrane [12], and administration of oxygen free radical scavengers reduces the occurrence of myringosclerosis in rat [13]. Nevertheless, myringosclerosis is an irreversible lesion once it occurs. Myringosclerosis over the whole tympanic membrane may severely impair mobility of the tympanic membrane and induce a mild to moderate hearing loss [14]. Thus, if possible, myringosclerotic deposits should be removed during myringoplasty. The present study describes the surgical management of myringosclerosis over a whole perforated tympanic membrane using simple underlay myringoplasty.

A previous study showed no significant difference in the success rate of myringoplasty between the myringosclerotic and the nonmyringosclerotic groups [15]. On the other hand, appropriate freshening of the perforation edges, with removal of sclerotic plaque, results in a high rate of successful closure of a perforated tympanic membrane with coexisting myringosclerosis [16]. Since both blood vessels and myringosclerosis are situated in the lamina propria of the tympanic membrane, blood supply to the graft may be blocked when myringosclerosis exists over a perforated tympanic membrane. When myringosclerosis is situated at the edge of a perforation, an otologist is tempted to remove the myringosclerosis. This induces tears in the residual tympanic membrane and a large perforation, which is technically more difficult to close if the myringosclerosis is removed together with epidermal and mucous layers. Our surgical technique, which preserves the epidermal layer and mucous layer as much as possible, does not create a larger perforation. In Japan, simple underlay myringoplasty with fibrin glue is a routine procedure with good results [9–11]. Therefore, removal of myringosclerosis at the edge of the perforation might be a beneficial technique for simple underlay myringoplasty in terms of the success rate and postoperative hearing threshold, especially when myringosclerosis extends over the entire tympanic membrane. However, there are no data to confirm that it improves either graft success or improves hearing thresholds because of no control group with myringosclerosis treated with underlaying technique without removal of plaque, with which they could have compared the results.

In conclusion, despite the small number of cases and having no control group, removal of myringosclerosis at the edge of a perforation is expected to improve graft success and a better-looking graft for simple underlay myringoplasty, especially when myringosclerosis extends over the entire tympanic membrane.
